# mGlu4R, mGlu7R, and mGlu8R allosteric modulation for treating acute and chronic neurodegenerative disorders

**DOI:** 10.1007/s43440-024-00657-7

**Published:** 2024-09-30

**Authors:** Helena Domin, Grzegorz Burnat

**Affiliations:** Maj Institute of Pharmacology, Department of Neurobiology, Polish Academy of Sciences, Smętna 12, Kraków, 31-343 Poland

**Keywords:** Neuroprotection, mGluR group III, mGluR dimer activation model, Allosteric ligands/Modulators, PAMs, NAMs

## Abstract

Neuroprotection, defined as safeguarding neurons from damage and death by inhibiting diverse pathological mechanisms, continues to be a promising approach for managing a range of central nervous system (CNS) disorders, including acute conditions such as ischemic stroke and traumatic brain injury (TBI) and chronic neurodegenerative diseases like Parkinson’s disease (PD), Alzheimer’s disease (AD), and multiple sclerosis (MS). These pathophysiological conditions involve excessive glutamatergic (Glu) transmission activity, which can lead to excitotoxicity. Inhibiting this excessive Glu transmission has been proposed as a potential therapeutic strategy for treating the CNS disorders mentioned. In particular, ligands of G protein-coupled receptors (GPCRs), including metabotropic glutamatergic receptors (mGluRs), have been recognized as promising options for inhibiting excessive Glu transmission. This review discusses the complex interactions of mGlu receptors with their subtypes, including the formation of homo- and heterodimers, which may vary in function and pharmacology depending on their protomer composition. Understanding these intricate details of mGlu receptor structure and function enhances researchers’ ability to develop targeted pharmacological interventions, potentially offering new therapeutic avenues for neurological and psychiatric disorders. This review also summarizes the current knowledge of the neuroprotective potential of ligands targeting group III mGluRs in preclinical cellular (in vitro) and animal (in vivo) models of ischemic stroke, TBI, PD, AD, and MS. In recent years, experiments have shown that compounds, especially those activating mGlu4 or mGlu7 receptors, exhibit protective effects in experimental ischemia models. The discovery of allosteric ligands for specific mGluR subtypes has led to reports suggesting that group III mGluRs may be promising targets for neuroprotective therapy in PD (mGlu4R), TBI (mGlu7R), and MS (mGlu8R).

**Graphical Abstract:**

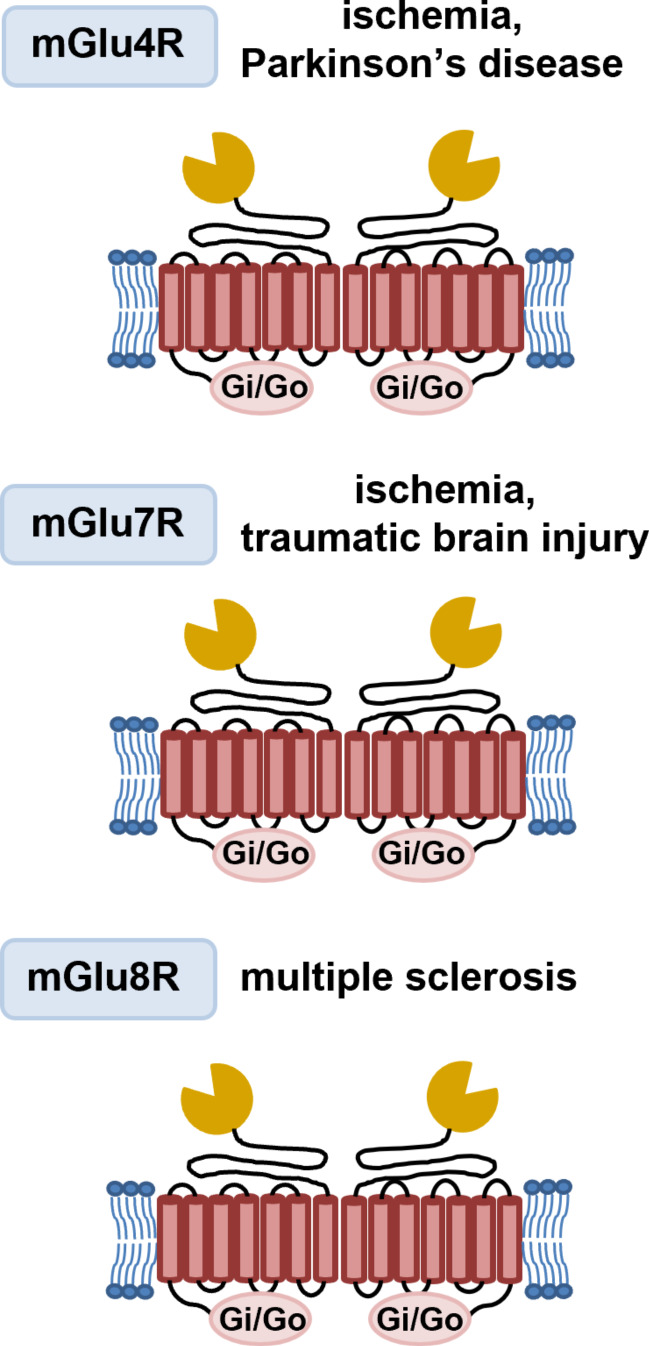

## Introduction

Neuroprotection refers to strategies and interventions aimed at preventing or slowing down the progression of neuronal damage or death caused by pathological processes associated with central nervous system (CNS) disorders, including acute ischemic stroke and traumatic brain injury (TBI), and chronic neurodegenerative diseases [1–9]. The goal of neuroprotection is to preserve the structure and function of neurons, thereby preventing or minimizing neurological deficits.

Regardless of the cause of neurodegeneration, in both acute (ischemic stroke and TBI) and chronic neurodegenerative diseases [Parkinson’s Disease (PD), Alzheimer’s Disease (AD), multiple sclerosis (MS)], a primary contributor to neuronal death is the excessive activity of glutamatergic (Glu) neurotransmission (excitotoxicity) [10–14]. Inhibiting this glutamate-dependent excitotoxicity could be one of the potential therapeutic strategies for treating these CNS pathologies.

According to the excitotoxicity hypothesis, a primary factor in the pathogenesis of neuronal damage is the elevated glutamate level in the extracellular space, which causes overstimulation of Glu receptors, primarily the ionotropic N-methyl-D-aspartate (NMDA)-subtype receptor. However, other subtypes of ionotropic glutamate receptors (iGluRs), including 2-amino-3-(3-hydroxy-5-methylisoxazol-4-yl) propionate (AMPA) and kainate (KA) receptors, have also been recognized for their crucial role in mediating excitotoxic neuronal cell death [15–17]. This overstimulation leads to energy depletion, ionic disturbances, and prolonged depolarization of neurons, initiating a pathophysiological cascade of reactions that may result in neuronal injury or death. This cascade of events causes a massive increase in the intracellular concentrations of calcium ions (Ca^2+^), triggering a series of harmful processes, including the activation of various enzymes (e.g., nitric oxide synthase, lipases, caspases, calpains, endonucleases), production of reactive oxygen species (ROS), formation of nitric oxide, and disruption of the structure and function of membranes, particularly those of the mitochondria and endoplasmic reticulum. Consequently, mitochondrial dysfunction, oxidative stress, and inflammation occur, leading to cell death through necrosis or apoptosis [18, 19].

**Fig. 1:**
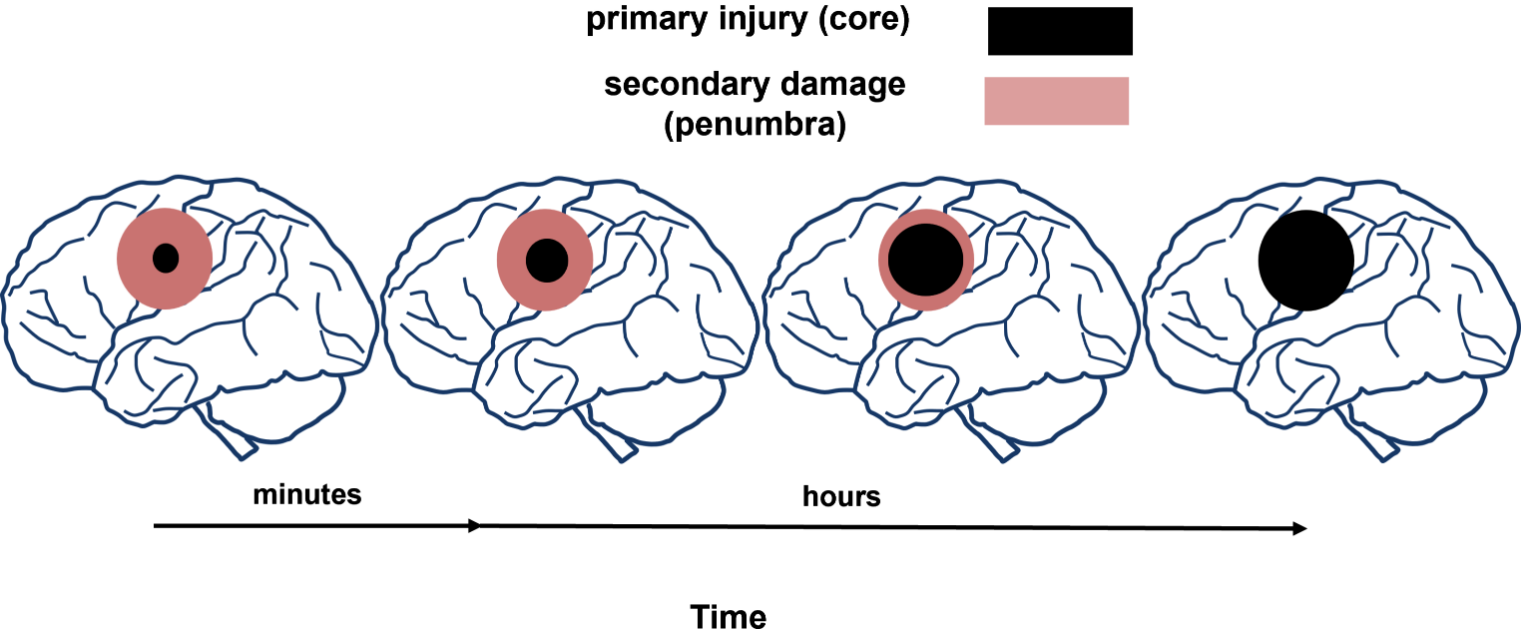
Schematic representation of brain damage process evolving over time. Neurotoxic events progress with specific dynamics and intensity. The area of secondary damage, known as the penumbra, surrounds the initial, more minor primary injury (referred to as the core) and expands within a few minutes to several hours

The processes described above, following excitotoxicity, occur with specific dynamics and intensity, depending on the nature of the injury, the time elapsed since its onset, and the location of the damaged area. This area of secondary damage, known as the penumbra, surrounds the initial, more minor primary injury (referred to as the core) and expands within a few minutes to several hours (Fig. 1), potentially serving as a target for therapeutic interventions [20, 21]. Since the therapeutic time window is crucial in establishing any neuroprotective compound’s potential clinical usefulness [22–24], finding effective agents, even several hours after the damaging factor, is significant for their use in clinical treatment.

Despite extensive experimental preclinical research on the role of excitotoxicity in various diseases and disorders using potential neuroprotective compounds, these efforts have failed to translate to clinical use for many reasons. Clinical trials using antagonists of iGluRs, primarily NMDA receptors, have been unsuccessful due to the narrow therapeutic time window, the occurrence of undesirable side effects such as psychosis, impairment of cognitive functions, memory and orientation, and movement disorders, the lack of efficacy, and high drug toxicity in humans preventing the use of doses that were effective for neuroprotection in rodents [8, 24–31].

Studies from the last three decades provide considerable evidence that ligands of G protein-coupled receptors (GPCRs), including metabotropic glutamate receptors (mGluRs), are particularly promising for inhibiting excessive glutamate (Glu) transmission. Because they are responsible for the modulation of excitation instead of participating directly in rapid synaptic transmission [32], they appear safer for therapeutic use than iGluR antagonists [33–39]. Based on the knowledge of the role of individual mGlu receptors in regulating cell excitability and synaptic transmission, the search for therapeutically effective mGluR ligands has focused on antagonists of group I mGlu receptors and agonists of group II and III mGlu receptors [40].

This review article is dedicated to the therapeutic potential of targeting group III mGluRs based on preclinical and clinical studies. The review is organized into four main parts. First, it focuses on the structural dynamics of mGlu receptors and allosteric modulation. The second part discusses the known subtypes and localization of group III mGluRs in various brain areas and at the subcellular level. The third part deals with the pharmacological tools available for these receptors, including orthosteric agonists and antagonists and allosteric ligands with agonist, PAM, and NAM activities. The fourth and last section summarizes our current knowledge of the effects of group III mGluR ligands in acute and chronic neurodegenerative disorders.

## Structural dynamics of mGlu receptors and allosteric modulation

Metabotropic glutamate receptors have been categorized into three groups based on their amino acid sequence homology, signal transduction pathways, and pharmacological profiles. Group I mGluRs, which include mGlu1R and mGlu5R, are positively linked to phospholipase C through the G_q_/G_11_ protein. When activated, these receptors stimulate the production of diacylglycerol (DAG) and inositol-1,4,5-triphosphate (IP_3_), mobilizing intracellular Ca^2+^ ions. Group II (mGlu2R and mGlu3R) and Group III (mGlu4R, mGlu6R, mGlu7R, and mGlu8R) mGluRs are negatively linked to adenylyl cyclase (AC) through G_i/o_ proteins. When activated, they inhibit the formation of cyclic adenosine monophosphate (cAMP) by interacting with G_αi/o_ [32, 41–44]. Group II and III mGlu receptors can also trigger pro-survival kinase pathways, such as MAPK/ERK (mitogen-activated protein kinase/extracellular signal-regulated kinase) and PI3-K/Akt (phosphatidylinositol-3-kinase/Akt), by interacting with G_αi/o_ proteins. This interaction promotes neuroprotection by generating neurotrophic factors [45–47].

Recently, it has been shown that mGluRs, including mGlu3R, mGlu7R, and mGlu8R, are capable of Glu-evoked desensitization and internalization (endocytosis) by GPCR kinases (GRKs) and β-arrestins (β-arrs, consisting of β-arr1 and β-arr2) [48–50]. Additionally, by interacting with other proteins, such as c-Jun N-terminal kinase 3 (JNK3) and MAPK/ERK, β-arrestins can facilitate alternative signaling pathways following agonist binding [51]. Thus, β-arrestin-mediated signaling pathways are part of the diverse signaling routes that can be activated depending on the agonist type, contributing to the concept of GPCR-biased agonism [52, 53]. Application of the concept of biased agonism (also referred to as “functional selectivity” or “ligand-biased signaling”) suggests the possibility of creating biased agonists as optimized therapeutics, which could offer enhanced effectiveness and/or fewer side effects [54, 55].

Notably, mGluRs phosphorylation by GRKs and beta-arrestin binding are not required for desensitization, and group 1 mGluRs endocytosis can occur in both agonist-dependent and -independent manners [56, 57]. It has been demonstrated that GRK2 contributes to the phosphorylation and desensitization of both mGlu1R and mGlu5R in human embryonic kidney (HEK 293) cells [58, 59]. However, GRK2 also contributes to the phosphorylation-independent desensitization of mGlu5R in primary mouse striatal neurons [60] and mGlu1R in HEK 293 cells [61]. As discussed by Iacovelli and colleagues, contrasting results obtained across different laboratories may arise not only from differences in experimental protocols and methods used to measure receptor activity but also from the cellular context in which receptor desensitization has been studied [62].

Metabotropic glutamate receptors share a similar overall structure due to their functional roles and common phylogenetic origin. The cDNA sequences encoding these receptors are relatively long, comprising over 2,500 nucleotides corresponding to approximately 850 amino acids. Sequence homology within receptor groups is around 70%, decreasing to about 45% between different mGlu receptor groups [63–65]. These structural features are characteristic of the entire receptor family rather than individual receptors, complicating the development of compounds with selectivity for specific receptors. Structurally, mGlu receptors belong to the C class of the GPCR protein family and function as obligatory dimers [66–68]. The extracellular portion of these receptors consists of just over 550 amino acids, forming a sizeable N-terminal domain known as the Venus Flytrap Domain (VFD), named for its resemblance to the carnivorous plant. This domain comprises two lobes positioned one above the other, with a cavity between them that serves as the orthosteric glutamate-binding site. Another extracellular domain is the cysteine-rich domain (CRD), which contains around 80 amino acids, nine of which are cysteine. The CRD links the VFD to the transmembrane portion and is responsible for signal transmission between the VFD and the intracellular part of the receptor. The third domain is a heptahelical sequence of over 250 amino acids, including extracellular and intracellular loops and seven transmembrane helical domain (7TMD) [69, 70]. This portion domain is crucial for interactions with G proteins and regulating the receptor’s activity. The final domain is the cytoplasmic C-terminal, which is medium-length with approximately 60 amino acids. This region is an essential regulatory site, facilitating interactions with intracellular signaling effectors [71].

As mentioned above, the orthosteric ligand-binding site is located in the extracellular portion formed by two lobes. It consists of two motifs: the proximal motif, conserved among mGlu receptors, and the distal motif, which is variable and allows selective ligand binding to specific mGlu receptor groups [72, 73]. The orthosteric ligand binds to a site in the extracellular VFD within a cavity between the two lobes of this receptor part. The two lobes are separated in the inactive form, allowing the orthosteric ligand to enter the cavity. Interaction of glutamate with amino acid residues in the binding pocket induces significant structural rearrangement, leading to local and global conformational conversion in the receptor structure.

Agonist binding brings the VFD lobes closer, activating the receptor and triggering appropriate effector proteins. This activation is relayed to the heptahelical transmembrane domain via the CRD, triggering intracellular signaling pathways. Interactions between cysteine residues in the CRD and VFD, forming disulfide bridges, appear crucial in this process. Mutations in the CRD can significantly weaken signal transmission to effector proteins despite the orthosteric ligand binding [74, 75].

**Fig. 2:**
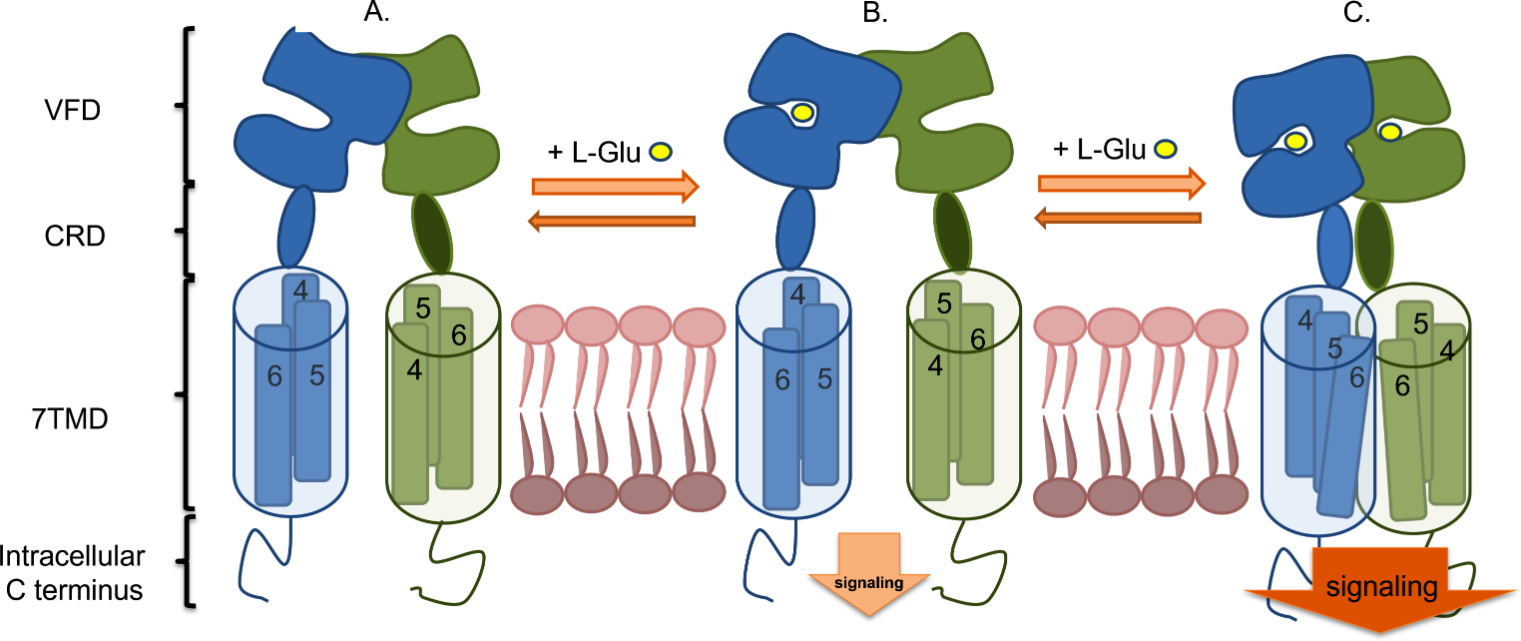
A simplified mGlu receptor dimer activation model and its domains: VFD - Venus Flytrap Domain; CRD - cysteine-rich domain; 7TMD - transmembrane heptahelical domain, shown as a cylinder with three highlighted transmembrane helices (TM 4–6) within the lipid bilayer and the intracellular C-terminus. The diagram presents three activation states of mGlu receptors, labeled **A-C**. The inactive state (**A**), called the open conformation, shows two protomers potentially close to each other due to possible interactions between helices TM4-5. State (**B**) illustrates the binding of the natural agonist glutamate to one of the subunits. This binding induces significant structural changes in the VFD, leading to the closure of the two lobes of this domain. Activation of one protomer may initiate signaling pathways, though to a limited extent, as indicated by the smaller vertical arrow. State (**C**) represents full dimer activation. In this state, substantial structural changes occur, leading to the complete activation of signaling pathways such as G_q/i_ protein activation. The saturation of agonist binding sites in the orthosteric site causes both parts of VFD to adopt a closed conformation and reorient relative to each other. The energy from these changes is transferred through the approaching rigid CRD sections, contributing to the rotation and approximation of the 7TMD. Consequently, TM6 helices interact with each other, stabilizing the fully active dimer structure, as depicted by the large vertical arrow. Additionally, transitions between receptor states can occur without activating compounds. The receptor adopts various conformations while maintaining equilibrium between transitions. The presence of an agonist shifts the equilibrium constant toward the active form, as illustrated by the horizontal arrows

Researchers distinguished several intermediate states of the receptor within each domain. Liauw and colleagues identified four states to describe receptor dynamics better, ranging from the inactive receptor state (stage 1) to full receptor activation (stage 4) in mGlu2 and mGlu3 receptors [76]. Without an agonist, the receptor can exist in intermediate states 1–4, where a dynamic equilibrium occurs. As the agonist concentration increases to saturate the binding sites thoroughly, this equilibrium shifts from the inactive state 1 through two intermediate states 2–3 to full receptor activation (stage 4) with extensive structural changes. Nevertheless, other receptor activation states are also observed, but with lesser frequency. The dynamics of transitions between states 4 and 3 have also been noted. According to the authors, state 3 corresponds to the agonist concentration at which the ligand caused half of the maximal response of the receptor, corresponding to the EC_50_ value [76]. The binding of the agonist not only causes the closure of the lobes of the orthosteric ligand-binding pocket but also consequently brings the VFD parts of the two protomers closer together through a few intermediate states associated with the open and closed forms of VFD [77]. Figure 2 presents the mGlu receptor dimer model and its activation process. To clarify, the agonist acts by stabilizing the closed conformation of the VFDs belonging to two protomers and shifting the equilibrium between the inactive and active orientations of the VFDs toward the active state, with increasing agonist concentration ultimately resulting in receptor activation.

Additionally, significant changes occur in the heptahelical region. Upon reorientation in the VFD, an active dimer, aided by the rigid CRDs, pushes both 7TMDs to reposition, causing them to rotate relative to the rest of the receptor. However, in the inactive state, the relative position of the two 7TMDs varies, with them facing each other through TM4 or TM5, either in contact or not. Receptor activation leads to a reorientation of the transmembrane helices, and this conformation is stabilized by the interaction between the two TM6 helices [78].

The second significant site related to the pharmacology of mGlu receptors and the synthesis of new ligands is the chloride binding pocket in the extracellular VFD adjacent to the glutamate binding site. Functional studies have demonstrated that the deprivation of chloride ions (Cl⁻) drastically decreases the activity of mGlu receptors, particularly those in group III [79]. Cl⁻ can act as an independent agonist through its orthosteric binding site or as a positive modulator of mGlu receptors, enhancing their potency [79, 80]. Among all mGlu receptors, the mGlu2R subtype from group II mGluRs is the least sensitive to the presence of this anion. Nonetheless, mGlu receptors are more susceptible to Cl⁻ than other class C GPCRs [79–81].

The allosteric site within the heptahelical transmembrane domain is the third critical site for regulating mGlu receptor activity. This site influences receptor engagement by binding an appropriate molecule and either enhancing or inhibiting its biological effects. In theory, compounds with modulatory properties were designed to alter the action of a selected receptor in the presence of an endogenous ligand by binding to a site different from the orthosteric site [82, 83]. Over time, additional groups of compounds have emerged. Besides the standard positive allosteric modulators (PAMs) and negative allosteric modulators (NAMs), there are ago-allosteric modulators (ago-PAMs), which enhance the binding of the orthosteric ligand and can also activate the receptor independently [82]. Allosteric agonists activate the receptor by binding to a site other than the orthosteric site.

In contrast, allosteric neutral ligands interact with allosteric sites without producing any biological effects associated with orthosteric ligands, competing with PAMs and NAMs for the binding site and thus inhibiting their effects [84]. These compounds are valuable research tools for studying mGlu receptors. Finally, inverse agonists inhibit the constitutive activity of the receptor, representing another critical group of compounds [85].

A crucial process following mGlu receptor activation by an orthosteric ligand is structural changes enabling physical interactions between receptor proteins, known as dimerization. Initially, mGlu receptors were thought to function exclusively as homodimers [86]. However, a subsequent study revealed the existence of heterodimeric mGluRs [68], with the authors noting that it is often unclear whether mGluRs are limited to forming strict heterodimers or if they result from the association of different homodimers or even higher-order homooligomers. Interestingly, 30 years ago, the mGluRs were assumed to be monomers [42, 63]; however, this was never directly demonstrated. Using biochemical and molecular techniques, Romano and colleagues later showed that mGluRs are not monomeric but form covalently linked dimers bound by disulfide bonds between conserved cysteines in the N-terminal extracellular domain [86]. Besides the disulfide bridge interactions in the VFD, the interface within the hydrophobic heptahelical domains is crucial for dimerization. Notably, in the inactive state, the TMDs of mGlu5R, mGlu7R, and mGlu2/7R do not interact with the opposing protomer, instead adopting an expanded conformation, where the extracellular domains keep the TMDs apart [87].

In contrast, for mGlu2R protomers, the TM5 region of one receptor interplays the TM4 of another receptor through hydrophobic interactions, as was mentioned above [78, 88]. The pharmacological properties of the receptors are strongly influenced by heterodimerization. Depending on the dimer composition, the agonist molecule may activate the system by binding to one subunit or only achieve activation when binding to both.

Similar observations apply to the action of allosteric modulators depending on the dimer composition [87]. PAMs or NAMs can be specific to homodimers, remaining inactive on heterodimers containing the same subunit, a phenomenon described by Moreno-Delgado and colleagues [89]. The authors compared the function of the mGlu2/4 heterodimer with that of the respective mGlu2 and mGlu4 homodimers. Remarkably, the receptors exhibited different potencies and maximum effects in response to synthetic ligands for group II and III mGlu receptors, depending on the configuration of the protomers. They also confirmed that both subunits are necessary for fully activating the mGlu2/4R dimer. Activating just one subunit by a specific ligand resulted in only partial heterodimer activation. In addition, the administration of individual PAMs had minimal effect on the heterodimer, in contrast to their co-application. For example, the authors observed the synergistic effect of selective mGlu2R PAM BINA and selective mGlu4R PAM VU0415374 on the mGlu2/4 heterodimer in the presence of glutamate at a concentration corresponding to EC_20_ for the mGlu2/4 heterodimer. Besides the experiments performed on transfected HEK 293 cell culture, Moreno-Delgado and colleagues also provided strong evidence of mGlu2/4 heterodimers in primary hippocampal neurons prepared from Sprague-Dawley rat embryos, highlighting the natural formation of these heterodimers in the brain [89].

Lin and colleagues observed similar findings in the in vitro and ex vivo studies that analyzed the activity of the mGlu7/8 heterodimer relative to the corresponding homodimers [90]. They found that two highly structurally related mGlu7R NAMs, VU6010608 and VU6010953, exhibited distinct activity profiles for the mGlu7/8 heterodimer. In the in vitro study conducted on HEK 293 cells, VU6010608 acted on both the mGlu7/7 homodimer and the mGlu7/8 heterodimer, whereas VU6010953 showed selectivity only for the mGlu7/7 homodimer. Additionally, an ex vivo study at the Schaffer collateral-CA1 (SC-CA1) synapses demonstrated the selective activities of these NAMs on the mGlu7/8 heterodimer [90]. These authors also describe the effects of two other mGlu7R NAMs, ADX71743 and MMPIP, which are active and selective for mGlu7 homodimer but exhibit different actions on the mGlu7/8 heterodimer. In the in vitro study, MMPIP showed no activity against this heterodimer, whereas ADX71743 inhibited its activity in the presence of the agonist L-AP4. The extension of the research to electrophysiological experiments using synapses from the Schaffer collateral-CA1 region showed no effect of MMPIP, which may suggest the presence of mGlu7R as a heterodimer in this region of the hippocampus.

As indicated by the presented information, mGlu receptors can dimerize both within and across different groups of mGluRs, further increasing the complexity of their interactions [91]. Another level of intricacy is presented in studies conducted by McCullock and colleagues, which analyzed the coupling of mGlu receptors with various G proteins [92]. However, the authors utilized homodimers in their research. The question in this context is how different G proteins couple with heterodimers.

Understanding these intricate details of mGlu receptor structure and function enhances researchers’ ability to develop targeted pharmacological interventions, offering potential therapeutic avenues for neurological and psychiatric disorders. In addition to preferences for homo- and heterodimerization among different mGlu receptor subtypes, there are also variations in the activation mechanisms of these receptor complexes.

To better understand the pharmacology of GPCRs, we need to consider receptor activation models that include orthosteric ligands and allosteric modulators. A fundamental version of these interactions is described by the two-state model, which qualitatively explains receptor behavior without considering further signaling pathways. This model presents the receptor in two possible conformations, active and inactive, between which it maintains equilibrium. Transitioning from one state to another involves conformational changes. Agonist binding to the receptor shifts this equilibrium towards the active form. In the two-stage model, an antagonist does not affect the equilibrium constant between the active and inactive states, binding equally to both forms. The antagonist prevents the agonist from binding to the orthosteric site and does not shift the equilibrium toward the activated receptor. Some receptors exhibit intrinsic activity where the equilibrium constant is moved towards the active form. A substance that inhibits such activity is called an inverse agonist.

This model seems simplified, especially regarding only two receptor forms, active and inactive. One iteration of this model is the allosteric two-state model with two binding sites. A more appropriate model includes multiple intermediate conformations, each recognizable by a specific compound. The interaction of a molecule with the receptor stabilizes one of many possible protein conformations.

## Background on group III mGluRs

Studies over the last decade indicate that group III metabotropic glutamate receptors may be a promising target for new neuroprotective drugs [40]. One of the most notable physiological effects of activating group III mGluRs, located presynaptically on glutamatergic and gamma-aminobutyric acid (GABA)ergic terminals, is the reduction of glutamatergic and GABA neurotransmission, respectively [32, 42, 93]. These receptors are distributed in various brain regions, except for mGlu6 receptors, located only in the retina [94]. A significant expression of the mGlu4 receptor subtype in rodent brains was observed in the molecular layer of the cerebellum and the granule cells of the olfactory bulb [95–97] as well as in globus pallidus (GP), a major component of the basal ganglia [97]. These authors pointed out that in the GP, mGlu4R is mainly localized presynaptically at symmetrical striatopallidal synapses of GABAergic neurons and, as a presynaptic heteroreceptor, may play an essential role in regulating GABA release from striatopallidal terminals [97].

Moderate mGlu4R immunoreactivity staining was found in the substantia nigra pars reticulata (SNpr) and the entopeduncular nucleus. Moderate to low mGlu4R immunoreactivity was present in the striatum, hippocampus, neocortex, and thalamus [97]. In the rat hippocampus, mGlu4R is found presynaptically at asymmetrical (likely glutamatergic) synapses and, as a presynaptic autoreceptor, may play a key role in regulating Glu release [64]. In addition to the presynaptic location of mGlu4R, the postsynaptic localization of this receptor subtype at asymmetrical synapses onto hippocampal pyramidal cells has been described [64].

Unlike the mGlu4 receptor, which is located presynaptically on both asymmetrical and symmetrical synapses, the mGlu7 receptor is presynaptically localized solely at asymmetrical synapses in the rat hippocampus, suggesting its presence only on glutamatergic terminals and its role as a presynaptic autoreceptor in influencing excitatory neurotransmission [64]. These authors also pointed to the postsynaptic mGlu7R localization both in the hippocampus and nonhippocampal regions, such as the striatum and GP [64, 98]. In addition, these authors observed high expression of mGlu7R on presynaptic terminals on rat striatum, GP, and SNpr [99]. Immunohistochemical and electron microscopy studies within rat basal ganglia revealed that mGlu7R is localized presynaptically on glutamatergic synapses of the corticostriatal pathway and, as an autoreceptor, is involved in regulating Glu release. It is also found presynaptically on terminals of GABAergic striatopallidal and striatonigral projections, where, as a heteroreceptor, it modulates GABA release [99].

In addition to the high expression of the mGlu7 receptor in the hippocampus and basal ganglia, they are also abundant in several other mammalian brain areas, including the olfactory system (the main and accessory olfactory bulbs, anterior olfactory nucleus, islands of Calleja, superficial layers of the olfactory tubercle), layer I of the neocortical regions, piriform and entorhinal cortex, superior colliculus, dorsal cochlear nucleus, amygdala (periamygdaloid cortex and amygdalohippocampal area), locus ceruleus, cerebellar nuclei, hypothalamic and thalamic nuclei and superficial layers of the medullary and spinal dorsal horns [98, 100].

Unlike other group III mGlu receptors located perisynaptically, mGlu7R is limited to the central presynaptic zone, specifically at the site of synaptic vesicle fusion [101]. Based on its location and the fact that this receptor subtype exhibits an extremely low affinity for Glu (requiring high µM to mM concentrations for activation) [102], it has been suggested that mGlu7R plays an essential role when Glu levels are elevated. This suggestion is supported by the findings that mice lacking the mGlu7 receptor (mGlu7R^−/−^) have increased seizure susceptibility [103]. Therefore, the mGlu7R subtype may serve as the brain’s evolutionary strategy to prevent pathological alterations in neuronal excitability and maintain homeostasis [104].

Compared to mGlu4R and mGlu7R, the CNS expression of the mGlu8 receptor is lower and more restricted [43]. Their strong expression has been found at presynaptic terminals in the olfactory bulb [105, 106], hippocampus [107, 108], striatum [109, 110] as well as in the pontine gray, lateral reticular nucleus of the thalamus, piriform, and entorhinal cortex [106, 111].

Group III mGlu receptors are expressed not only in neurons but also in glial cells. The presence of mGlu4 receptors has been observed on both astrocytes and microglia in cultured cells [112–114] and in oligodendrocytes [114]. According to Spampinato and colleagues, oligodendrocytes express mGlu4 receptors exclusively during early stages of maturation (O4-positive) but not during later stages of differentiation (myelin basic protein, MBP-positive) [114]. mGlu6 receptors are found on astrocytes and microglia in cultured cells [112]. mGlu7 receptors have been observed in astrocytes in cultured cells [113, 115]. The expression of mGlu8 receptors is present in cultured microglia [112]. Additionally, strong mGlu8R immunoreactivity has been found in reactive microglia/macrophages during demyelination in MS patients [116]. mGlu8R expression has also been detected in human reactive astrocytes in chronic active MS lesions [116].

## Pharmacological agents targeting group III mGluRs

### Group III mGluR orthosteric ligands

The natural compound activating group III mGlu receptors is L-glutamic acid, which interacts with the binding pocket, leading to conformational changes, dimerization, and subsequent receptor activation and secondary messenger signaling. The glutamate binding pocket is evolutionarily conserved within mGlu receptors [117]. Consequently, the first agonistic compounds were structurally similar to L-Glu. In most cases, these compounds showed selectivity for one receptor group rather than specific receptors. Another endogenous orthosteric agonist is L-serine-O-phosphate (L-SOP). This compound, a direct precursor of L-serine, is produced by the enzyme phosphoserine aminotransferase (PSAT) and metabolized to L-serine by phosphoserine phosphatase (PSP). L-SOP is a non-selective endogenous orthosteric agonist of group III mGlu receptors and also exhibits antagonistic properties towards mGlu1 and mGlu2 receptors [118, 119].

One of the first compounds with confirmed selectivity for group III mGlu receptors was L-2-amino-4-phosphonobutyric acid (L-AP4). In 1985, Slaughter and Miller described the effect of this glutamate analog, which inhibited the light response of bipolar eye cells [120]. L-SOP has an almost identical steric structure to L-AP4. L-SOP is mainly dianionic at physiological pH, while a significant portion of L-AP4 is monoanionic. Based on these two compounds, antagonistic molecules were also developed by replacing one of the hydrogens on the amine group with a methyl group, producing 2-amino-2-methyl-4-phosphonobutyrate (MAP4) from L-AP4 and a-methyl-serine-O-phosphate (MSOP) from L-SOP [121, 122].

Another group of compounds was derived from phenylglycine. Structural analogs include the mGlu4/8 receptor agonist 4-phosphonophenylglycine [(*R*,* S*)-PPG] and mGlu8 receptor agonist (S)-3,4-Dicarboxyphenylglycine [(*S*)-3,4-DCPG)] [123, 124]. Phenylglycine antagonists of mGluRs have also been identified, including (RS)-α-methyl-4-phosphonophenylglycine glycine (MPPG) and (RS)-alpha-cyclopropyl-4-phosphonophenylglycine (CPPG) [125, 126]. The first generation of compounds, including L-AP4, L-SOP, and (*R*,* S*)-PPG, had negligible properties for crossing the blood-brain barrier (BBB) due to the presence of phosphate groups. The mentioned (*S*)-3,4-DCPG, lacking this group, improved its pharmacological properties.

The first study showing that orthosteric agonist of group III mGluRs can cross the BBB after intraperitoneal (*ip*) administration in rats was conducted by Palucha-Poniewiera and colleagues using (1 S,3R,4 S)-1-aminocyclopentane-1,2,4-tricarboxylic acid], ACPT-I [127]. ACPT-I is one of the four ACPT stereoisomers developed from the group I and group II mGlu receptor agonist, (1 S,3R)-1-Aminocyclopentane-1,3-dicarboxylic acid [(1 S,3R)-ACPD], by adding a third carboxylic group at position 4 in the cyclopentane ring [128]. ACPT-I has the highest potency for mGlu4R over mGlu8 and mGlu7 receptors without significant effects on other mGlu receptor groups or iGluRs [128, 129].

The next generation of agonistic compounds begins with 3-amino-3-carboxypropyl-20-carboxyethylphosphinic acid (PCEP), which is based on the glutamate analog L-AP4 and features an extended carbon tail reaching beyond the glutamate binding pocket [130, 131]. This design modification aims to create more selective compounds targeting the less conserved region of the VFD. Further chemical optimization produced analogs such as [((3 S) − 3-Amino-3-carboxy)propyl][(4-hydroxy-5-methoxy-3-nitrophenyl)hydroxymethyl]phosphinic acid (LSP1-2111) and (2 S) − 2-amino-4-({[4-(carboxymethoxy)phenyl](hydroxy)methyl} (hydroxy)phosphoryl)butanoic acid (LSP4-2022), which exhibit increased selectivity for mGlu4R over another subtypes of group III receptors and no effect on other mGluRs groups [131]. These compounds were designed considering the previously mentioned chloride binding pocket near the N-terminal glutamate binding site. A list of mGluR III group orthosteric agonists is described in Table 1.

**Table 1:** Orthosteric and allosteric agonists of group III mGlu receptors

No.	Compound name	mGlu4R EC_50_ (µM)	mGlu7R EC_50_ (µM)	mGlu8R EC_50_ (µM)	Comments	Reference(s)
1	L-Glu	3–20	670	2.5–11	endogenous ligand, non-selective over mGluR	[196, 197]
2	L-AP4	0.1–1.2	160–500	0.06–0.6	agonist of group III mGluR	[196, 198]
3	L-thioAP4	0.039	197	0.054	selective agonist of group III mGluR	[199]
4	L-SOP	1–4	31-1200	0.3–1.8	endogenous ligand, non-selective over mGluR	[196]
5	S-PPG	5.2	185	0.2	mGlu4/8R agonist	[200]
6	(*S)*-3,4 DCPG	8.8	> 100	0.031	selective mGlu8R agonist	[201]
7	ACPT-I	1.7–7.2	280–1200	5.13–10.1	agonist of group III mGluR	[198, 201]
8	PCEP	5.9	89	6.5	interact with Cl^−^ pocket	[131]
9	LSP1-2111	0.9–2.2	19.4–53	53–101	interact with Cl^−^ pocket	[131, 198]
10	LSP4-2022	0.11	11.6	29.2	interact with Cl^−^ pocket	[131]
11	AMN082	-	0.064–0.29	-	allosteric agonist	[138]
12	CVN636	-	0.002	-	allosteric agonist	[139]

(-) not active according to the references

Among the compounds inhibiting group III mGluRs activity mentioned earlier, such as MSOP and MAP4, there are a few noteworthy ones, especially for their contribution to understanding the glutamatergic system’s function and pathophysiology. Generally, these compounds do not usually exhibit selectivity for a specific receptor but rather for the entire group III mGlu receptors. One exception is 7-hydroxy-3-(4-iodophenoxy)-4 H-chromen-4-one (XAP044), the first selective mGlu7R antagonist that binds within the extracellular VFD, close to the orthosteric agonist L-glutamate binding site [132]. This compound’s structure is unrelated to other ligands for mGluRs and was discovered through high-throughput screening (HTS) and lead optimization. The authors observed that XAP044 demonstrated full antagonist activity at mGlu7R, similar to classical L-glutamate-site blockers like MPPG or CPPG, which, unlike XAP044, do not differentiate between the four group III mGluR subtypes [132].

Interestingly, Cristiano and colleagues recently reported that XAP044 does not block the Glu binding site directly but prevents glutamate from closing the VFD [133]. In other words, XAP044 inhibits the receptors’ function without occupying the glutamate binding site itself, and this mechanism highlights XAP-044’s unique mode of action as a selective mGlu7R antagonist. Interestingly, although XAP044 does not act via the seven-transmembrane region but through a binding pocket located in the extracellular VFD of mGlu7R, a region typically associated with orthosteric ligand binding, some authors have described XAP044 as a selective mGlu7R NAM [134], a mGlu7R allosteric modulator [133] or an orthosteric-like mGlu7R antagonist [135].

The above-mentioned phenylglycine antagonist of mGluRs, CPPG, is a group II/III antagonist with approximately 20-fold selectivity for group III over group II mGluRs (IC_50_ values of 2.2 and 46.2 nM, respectively) and exhibits weaker antagonist activity against the group I mGluRs [126]. It was derived from α-methyl-4-carboxyphenylglycine (MCPG), a non-selective mGluR ligand, by replacing the 4-carboxy group with a 4-phosphono and cyclopropyl group [126].

It is also worth mentioning the compound developed by Lilly Research Laboratories, (2 S)-2-Amino-2-[(1 S,2 S)-2-carboxycycloprop-1-yl]-3-(xanth-9-yl) propanoic acid (LY341495) [136]. This molecule was created as one of the analogs of (1 S,1′S,2′S)-carboxycyclopropylglycine (L-CCG), an agonist of group II mGlu receptors. In addition to its antagonistic properties against group II receptors, LY341495 inhibits group III receptors with slightly lower efficiency [137]. Table 2 summarizes existing orthosteric group III mGluR antagonists.

**Table 2:** The orthosteric antagonist of group III mGlu receptors

No.	Compound name	mGlu4R IC_50_ (µM)	mGlu7R IC_50_ (µM)	mGlu8R IC_50_ (µM)	Comments	Reference(s)
1	ACPT-II	77–125	-	123	the non-selective antagonist of group II/III mGluR	[196]
2	MAP4	90–190	-	25–105	antagonist of group III mGluR	[196]
3	MPPG	54–500	300	20–50	group III over group II mGluR antagonist	[196]
4	LY341495	22	0.99	0.17	non-selective antagonist mGluRs	[196]
5	XAP044	-	2.8–3.5	33	potent mGlu7R antagonist	[132]
6	DCG-IV	22	25–40	15–32	agonist of group II mGluR; antagonist of group III mGluR	[196]

(-) not active according to the references

Most compounds interacting with the glutamate binding pocket, including agonists and antagonists, do not exhibit high selectivity. Using the chloride binding site has improved selectivity for specific mGlu receptors.

### Group III mGluR allosteric ligands

Another group of agonists interacts outside the glutamate binding pocket and is therefore termed allosteric agonists. Among group III mGluRs allosteric agonists, N,N-dibenzhydrylethane-1,2-diamine dihydrochloride (AMN082) and (S)-2-(4-fluorophenyl)-N-((3 S,4 S)-4-(methylsulfonyl)chroman-3-yl)propanamide (CVN636) are most prominent, both selectively activating mGlu7 receptor [138, 139]. AMN082 was identified through high-throughput random screening of chemical libraries. Its structure does not resemble other compounds interacting with mGluR group III. Mitsukawa and colleagues showed through studies on chimeric mGlu7-6 receptors that AMN082 interacts with the transmembrane part of mGlu7 [138]. It is speculated that this molecule binds to both subunits of the mGlu7R homodimer at the C-terminal or intracellular loops of the 7TM domain without needing the presence of an orthosteric agonist. Another allosteric mGlu7 receptor agonist, CVN636, was discovered through HTS and lead structure optimization [139]. CVN636’s activity is not blocked by the non-specific mGluR antagonist LY341495, suggesting its allosteric action. Unlike AMN082, CVN636 does not exhibit off-target effects and does not cause receptor desensitization. CVN636 treatment reduced ethanol self-administration in Marchigian Sardinian (msP) rats trained to self-administer alcohol, indicating its ability to cross the BBB [139]. Examples such as AMN082 and CVN636 demonstrate the effectiveness of strategies identifying compounds binding to allosteric sites independent of the natural ligand binding sites. Table 1 summarizes the currently available group III mGluR allosteric agonists.

### Group III mGluR positive allosteric modulators (PAMs)

N-Phenyl-7-(hydroxyimino)cyclopropa[b]chromen-1a-carboxamide (PHCCC) was the first modulator selective for group III receptors, specifically mGlu4R. Interestingly, this compound was first described in 1996 by Annoura and colleagues as a mGlu1R antagonist [140]. In 2003, the other authors demonstrated its PAM properties for the mGlu4 receptor [141]. Notably, PHCCC is structurally similar to 7-hydroxyiminocyclopropan[b]chromen-1a-carboxylic acid ethyl ester (CPCCCEt), a non-amino acid antagonist of the mGlu1 receptor (actually NAM for mGlu1R) [142]. Another compound, N-Phenyl-7-(hydroxyimino)cyclopropa[b]chromen-1a-carboxamide (MPEP), also exhibits dual activity; previously known as a non-competitive mGlu5 antagonist, it was later described by Mathiesen and colleagues as mGlu4R PAM [143].

Since PHCCC exhibited low water solubility, poor bioavailability, and affinity for the mGlu1 receptor, its usefulness as a PAM for mGlu4R was limited [144]. Over recent years, significant progress has been made in the search for more selective PAMs of group III mGlu receptors. Among these compounds are synthesized mGlu4R PAMs, such among others as cis-2-[[(3,5Dichlorophenyl)amino]carbonyl]cyclohexanecarboxylic acid (VU0155041), (1 S, 2R)-N-(3,4-dichlorophenyl)-cyclohexane-1,2-dicarboxamide (LU AF21934), N-(Chloro-3-methoxyphenyl)-2-picolinamide (VU0361737) and N-{6-[3-(morpholin-4-yl)propyl]-2-(thieno[3,2-c]pyridin-6-yl)-4 H-1-benzopyran-4-ylidene}hydroxylamine (PXT002331, actually foliglurax), mGlu8R PAM 2-[[(4-Bromophenyl)methyl]thio]-N-[4-(1-methylpropyl)phenyl]acetamide (AZ012216052) and mGlu7/8R PAM 3-(2,3-Difluoro-4-methoxyphenyl)-2,5-dimethyl-7-(trifluoromethyl)pyrazolo[1,5-a]pyrimidine (VU6005649) [144–149].

VU0155041 demonstrated significant mGlu4R PAM activity and showed partial agonist activity at mGlu4R at a place different from the orthosteric binding site, indicating that this compound is a mixed allosteric agonist/PAM of mGlu4R [144]. VU0155041 significantly improves aqueous solubility compared to PHCCC [144]; however, its weak bioavailability limits its therapeutic usefulness [150]. mGlu4R PAMs such as LU AF21934, VU0361737, and foliglurax improved their PAM potency, reduced agonistic activity, and were centrally penetrating compared to VU0155041 [145–147]. Foliglurax, based on its favorable and unique preclinical profile, was selected as a candidate for clinical trials [151, 152].

For mGlu7 and mGlu8 receptors, achieving selectivity remains challenging. Both VU6005649 and AZ012216052 are not considered selective. VU6005649 is described as a mGlu7 PAM with an EC_50_ of 0.6 µM for mGlu7R and 2.6 µM for mGlu8R [149]. Similarly, AZ012216052 is described as a PAM for mGlu8R [148]. However, in another study, these authors suggested that the effects of AZ012216052 involve receptors other than mGlu8R, most probably mGlu4R [153]. In addition, other authors also observed activity AZ122160052 towards mGlu4R as well as mGlu5R [154]. A list of group III mGluR PAMs is outlined in Table 3.

**Table 3:** Positive allosteric modulators (PAMs) of group III mGlu receptors

No.	Compound name	mGlu4R EC_50_ (µM)	mGlu7R EC_50_ (µM)	mGlu8R EC_50_ (µM)	Comments	Reference(s)
1	ADX88178	0.0035	-	2.2	mGlu4/8R PAM	[202]
2	AZ12216052	n.r.	n.r.	1	mGlu8R PAM	[148]
3	Lu AF21934	0.5	n.r.	n.r.	mGlu4R PAM	[145]
4	PHCCC	2.8	-	-	mGlu4R PAM, mGlu1R antagonist	[140, 141]
5	PXT002331 (foliglurax)	0.078	n.r.	n.r.	mGlu4R PAM	[147]
6	SIB183	0.15–1.08	-	-	mGlu4R PAM, mGlu5R antagonist	[143]
7	VU0155041	0.79 (2.5 as partial agonist)	-	-	mGlu4R PAM, partial agonist of mGlu4R	[144]
8	VU0361737 (ML-128)	0.008–0.22	-	week agonist, 2.7 fold shift of L-Glu concentration-response curve	mGlu4R PAM	[146]
9	VU6005649	> 10	0.65	2.6	mGlu7/8R PAM	[149]
10	VU6027459	> 10	1.6	> 10	mGlu7R PAM	[203]
11	VU0422288	0.001	0.0014	0.0012	mGluR III group PAM	[203, 204]
12	VU6046980	> 30	0.15	> 30	mGlu7R PAM	[205]
13	Optogluram-2	0.008	n.r.	n.r	photoswitchable mGlu4R PAM	[206]

n.r., not reported; (-)not active according to the references; (> ) more than

### Group III mGluR negative allosteric modulators (NAMs)

In contrast to PAMs, significantly fewer compounds act as NAMs for group III mGlu receptors. The availability of NAMs inhibiting the activity of group III receptors, especially mGlu4R and mGlu8R, is limited. However, several selective compounds with good pharmacological properties have been identified for mGlu7R. A notable NAM is OptoGluNAM4.1, which contains an azobenzene group that undergoes conformational changes under blue light, leading to light-dependent biological activity [155]. OptoGluNAM4.1 has been used in vivo in zebrafish and mouse models of chronic pain, though in vitro results confirming high selectivity for mGlu4 over other mGluRs are lacking.

The first selective NAM for mGlu7, (6-(4-Methoxyphenyl)-5-methyl-3-(4-pyridinyl)-isoxazolo[4,5-c]pyridin-4(5 H)-one) (MMPIP), was followed by (6-(2,4-dimethylphenyl)-2-ethyl-6,7-dihydrobenzo[d]oxazol-4(5 H)-one) (ADX71743) a molecule with high specificity and allosteric inhibitory activity [156, 157]. Both compounds enhance forskolin-stimulated cAMP production dose-dependently in T-REx 293 cells overexpressing human mGlu7 receptors, suggesting an inverse agonist features component [158].

Another promising group of NAMs includes the quinazoline-based compound (2-(2-Chlorophenyl)-6-(2,3-dimethoxyphenyl)-3-methylquinazolin-4(3 H)-one) (ALX-171) has been recently studied for its selectivity in vitro and pharmacokinetics in vivo [159]. Although it has slightly lower potency than MMPIP and ADX71743, ALX-171 exhibits good brain penetration and significantly advances the development of selective NAMs for mGlu7R [159]. Group III mGluR NAMs are listed in Table 4.

**Table 4:** Negative allosteric modulators (NAMs) of group III mGlu receptors

No.	Compound name	mGlu4R IC_50_ (µM)	mGlu7R IC_50_ (µM)	mGlu8R IC_50_ (µM)	Comments	Reference(s)
1	ADX71743	-	264–630	-	mGlu7R NAM	[157]
2	ALX-171	-	6.14	-	mGlu7R NAM	[159]
3	MMPIP	-	0.026	-	mGlu7R NAM	[156]
4	OptoGluNAM4.1	n.r.	n.r.	-	photoswitchable mGlu4R NAM, partial mGlu7R NAM	[207]
5	VU6010608	> 10	0.76	> 10	mGlu7R NAM	[208]
6	VU6012962	> 10	0.035	> 10	mGlu7R NAM	[209]

n.r., not reported; (-)not active according to the references; (>) more than

## Neuroprotective potential of the group III mGlu receptor orthosteric agonists in acute and chronic neurodegenerative brain damage: involvement of glutamatergic inhibition

Preclinical studies in cellular (in vitro) and animal (in vivo) models over the past couple of decades indicate a significant role of group III mGluRs in neuroprotection. First, reports revealing the neuroprotective potential of compounds activating group III mGlu receptors emerged from using non-selective orthosteric ligands (L-AP4, L-SOP, (*R*,* S*)-PPG) in various in vitro and in vivo models of excitotoxicity induced by neurotoxins [46, 102, 160–163]. Bruno and colleagues suggested that the mGlu4 receptor plays a role in neuroprotection in both in vitro and in vivo models of excitotoxicity evoked by NMDA [161]. Their in vitro research demonstrated that L-AP4, L-SOP, and (*R*,* S*)-PPG provided neuroprotection against NMDA-induced excitotoxicity in cortical cultures from wild-type mice (mGlu4R^+/+^) but were ineffective in mGlu4R-deficient (mGlu4R^−/−^) cultures. Additionally, they found that cortical cultures prepared from mGlu4R-deficient mice were more susceptible to NMDA-induced excitotoxicity than wild-type cultures. Their in vivo experiments demonstrated that low concentrations of (*R*,* S*)-PPG were neuroprotective in wild-type mice but not in mice lacking the mGlu4R. However, higher concentrations of (*R*,* S*)-PPG were effective in both strains [161]. These authors postulated that a possible mechanism underlying the neuroprotective effects of L-AP4 or (*R*,* S*)-PPG was related to reduced NMDA-evoked extracellular Glu levels [161].

In most studies mentioned above, the orthosteric agonists of group III mGlu receptors were administered before, concomitantly, or immediately after the excitotoxic injury. Importantly, our research demonstrated that ACPT-I provided neuroprotection against KA-induced excitotoxicity both in vitro (using primary mouse cortical and hippocampal neuronal cultures) and in vivo (in the rat hippocampus), even when given 3 h post-KA exposure [164]. The neuroprotective effects of ACPT-I observed in the in vitro study were blocked by CPPG [164]. We suggested, among others, that the mechanism of neuroprotective action of ACPT-I against excitotoxicity evoked by KA was connected with the inhibition of Glu-release [164]. In a follow-up study using an in vitro excitotoxic neuronal cell injury model, ACPT-I demonstrated neuroprotective effects against Glu-induced damage in primary mouse hippocampal neuronal cultures [165].

The favorable neuroprotective efficacy of ACPT-I was also observed in our studies in the in vitro and in vivo ischemic models [166, 167]. The neuroprotective potential of ACPT-I in primary cultures of mouse cortical neurons exposed to 3 h of oxygen-glucose deprivation (OGD) was evident 30 min after the end of OGD [166]. We suggested that the mGlu4 receptor activation protected cortical neurons from ischemic injury, as we observed a synergism in the neuroprotective effect of low non-effective concentrations of ACPT-I and mGlu4R PAMs [166]. In this in vitro study, we also found that ACPT-I reduced OGD-induced glutamate release, suggesting that the neuroprotective effect of ACPT-I may involve excessive glutamatergic inhibition [166].

In the in vivo study, ACPT-I provided neuroprotection to both healthy normotensive Sprague Dawley rats and spontaneously hypertensive rats (SHR) that underwent transient middle cerebral artery occlusion/reperfusion (MCAO/R) [166, 167]. In both normotensive rats and rats with essential hypertension, ACPT-I not only reduced the MCAO-induced cortico-striatal damage but also improved postischemic gait disturbances and motor deficits when administered 30 min after the start of MCAO or 30 min after the start of reperfusion [166, 167]. In addition, in SHR, ACPT-I had a beneficial effect on sensory and tactile functions when injected 30 min after starting MCAO [167].

The neuroprotective potential of the group III mGlu receptor orthosteric agonists in acute neurodegeneration was also observed in cellular and animal models of TBI. In an in vitro model of TBI, two orthosteric agonists of group III mGluR (L-AP4 and L-SOP) reduced TBI-induced neurotoxicity in rat mixed neuronal/glial cultures in a similar concentration-dependent fashion [168]. In this study, L-AP4 and L-SOP effectively reduced TBI-induced cell death when administered 30 min before the injury, with treatment continuing for 16–18 h post-injury. Conversely, treatment with group III mGluR orthosteric antagonists MAP4 or MSOP worsened the traumatic injury, an effect that was mitigated by L-SOP and L-AP4 [168].

In the in vivo model of TBI, in this case, it was diffuse brain injury (DBI), L-AP4 protected neurons, and improved motor and cognitive functions induced by DBI in rats [169, 170]. Contrary to these results, the other group III mGluR orthosteric agonist (*R*,* S*)-PPG showed no neuroprotective effect in an in vivo rat model of TBI [171].

The neuroprotective potential of group III mGluR orthosteric agonists such as L-AP4, L-SOP, (*R*,* S*)-PPG, and ACPT-I has also been reported in chronic neurodegenerative brain damage in both in vitro and in vivo models. In the in vitro model, using primary cultures of rodent cortical cells, L-AP4, L-SOP, and (*R*,* S*)-PPG were protective against the beta-amyloid peptide (βAP)-induced neurotoxicity, which serves as a cellular model of Alzheimer’s disease (AD) [172–174]. In primary cultures of microglia, L-AP4 and (*R*,* S*)-PPG reduced microglial reactivity activated by βAP; however, these orthosteric ligands did not provide neuroprotection against βAP-induced microglial neurotoxicity [112]. In a cellular model of Parkinson’s disease (PD) using human neuroblastoma SH-SY5Y cells, neuroprotective effects of group III mGluR orthosteric agonists such as ACPT-I and (*S*)-3,4-DCPG were observed against the dopaminergic mitochondrial neurotoxin, 1-methyl-4-Phenylpyridinium ion [MPP(+)] [175].

In the rat model of PD, subchronically and acute treatment with L-AP4 displayed neuroprotective activity against parkinsonian toxin 6-hydroxydopamine (6-OHDA)-induced neurotoxicity. L-AP4 protected the nigrostriatal system at histological and neurochemical levels against the neurotoxic effects of 6-OHDA [176–178] and improved motor function impaired by 6-OHDA [178]. These authors suggested that the possible mechanism underlying the neuroprotective effect of L-AP4 might be related to the inhibition of glutamate release in the SN [178].

## The role of mGlu4, mGlu7, and mGlu8 receptors in acute and chronic neurodegenerative disorders

### mGlu4 receptor

#### Ischemia

In the in vivo models, mGlu4R PAM PHCCC provided neuroprotection against ischemic brain damage in mice subjected to permanent MCAO and in rats subjected to endothelin-1 (Et-1)-induced transient focal ischemia [179]. PHCCC diminished MCAO-induced infarction volume and enhanced postischemic sensorimotor function when administered 30 min before ischemia in mice and 20 min after Et-1 infusion in rats. Interestingly, PHCCC was ineffective in mGlu4R-deficient (mGlu4R^−/−^) mice, which were more susceptible to ischemic damage compared to wild-type mice (mGlu4R^+/+^), suggesting that endogenous activation of mGlu4 receptors helps limit the extent of ischemic neurodegeneration [179]. These authors postulated that the activation of mGlu4R by PHCCC might reduce excessive glutamate release and thus limit ischemia-induced excitotoxicity.

In our in vitro ischemic model, PHCCC and other mGlu4R PAM VU0155041 protected primary cultures of mouse cortical neurons against the OGD-evoked neurotoxicity [166]. In a subsequent in vitro study, Zhang and colleagues confirmed the neuroprotective activity of VU0155041 against OGD-induced injury in primary cultures of human neural stem cells (hNSCs) from the human fetus cortex [180].

### Neurodegenerative disorders

The neuroprotective potential of mGlu4R PAM, PHCCC, has also been reported in a mouse model of PD using the parkinsonian toxin 1-methyl-4-phenyl-1,2,3,6-tetrahydropyridine (MPTP) [181]. PHCCC administered subcutaneously (*sc*) or *ip* 30 min before the toxin protected the nigrostriatal neurons [substantia nigra pars compacta (SNpc), striatum] against MPTP-induced neurotoxicity. Interestingly, PHCCC was neuroprotective in wild-type mice but was ineffective in mGlu4R^−/−^ mice [181]. The protective effects on nigrostriatal neurons were also observed following the administration of PHCCC into the external GP. These authors suggested that the neuroprotective effect of PHCCC might be due to the activation of mGlu4 receptors, which reduces GABA release in the external GP. This reduction leads to the disinhibition of GABAergic neurons projecting from the external GP to the subthalamic nucleus, inhibiting glutamatergic neurons in the subthalamic nucleus that project to the internal GP and SNpc. Additionally, they postulated that the inhibition of glutamate release in the SNpc substantia nigra pars compacta is mediated by the presynaptic mGlu4 receptor localized in this structure [181].

In another study using a rat model of PD induced by the 6-OHDA, mGlu4R PAM, VU0155041 protected tyrosine hydroxylase immunoreactive (TH-ir) neurons against 6-OHDA-induced neurotoxicity in SNpc. In addition, VU0155041 improved motor function impaired by 6-OHDA [182].

The neuroprotective potential of the mGlu4R PAMs in chronic neurodegeneration has also been found in cellular models. In the in vitro model of AD, PHCCC protected primary mixed cultures of mouse cortical cells against βAP-induced neurotoxicity [141]. In the in vitro model of PD, VU0361737 provided neuroprotection against the neurotoxin MPP(+) in undifferentiated (UN) human neuroblastoma (UN-SH-SY5Y) cells [175].

Further preclinical experiments conducted on non-human primates have confirmed the favorable efficacy of mGlu4R positive allosteric modulation using mGlu4R PAM PXT002331 (foliglurax) in antiparkinsonian effects [183]. These authors demonstrated that foliglurax, as an adjunct to L-DOPA therapy, effectively and in a dose-dependent manner reversed parkinsonian motor symptoms in macaques, such as bradykinesia, tremors, posture issues, and mobility. In addition, foliglurax significantly reduced the severity of dyskinesia, demonstrating therapeutic efficacy against both Parkinson’s motor impairments and L-DOPA-induced dyskinesia. However, in clinical trials of a randomized, double-blind, controlled phase II study, foliglurax, despite showing a dose-dependent trend, was ineffective in alleviating L-DOPA-induced motor complications in patients with Parkinson’s disease [152]. Recent studies in both rodents (rats) and non-human primates (marmosets) confirmed the lack of efficacy of mGlu4R positive allosteric modulation using mGlu4R PAMs such as Lu AF21934 and ADX88178 (the latter being a mGlu4/8R PAM, see Table 3) in reducing L-DOPA-induced dyskinesia [184].

Regarding foliglurax, a recent study first demonstrated its neuroprotective potential in a mouse model of Parkinson’s disease using the toxin MPTP [185]. These findings showed that orally administered foliglurax protected striatal dopamine (DA) neurons from MPTP toxicity and reduced MPTP-induced activation of astrocytes in the striatum, suggesting a possible anti-inflammatory mechanism underlying its neuroprotective effect. The authors rightly pointed out that the exclusive use of male mice was a limitation of their experiments. They emphasized the need to expand the experiments to include ovariectomized female mice to approximate better the clinical situation, where Parkinson’s patients are often older women with menopausal hormonal status.

### mGlu7 receptor

#### Ischemia

For the first time, the neuroprotective potential of mGlu7R allosteric agonist AMN082 against ischemic injury was observed in primary cultures of mouse cortical neurons exposed to 3 h of OGD [186]. Our findings demonstrated that AMN082 provided neuroprotection against OGD-evoked neurotoxicity even when administered 30 min after the end of ischemic damage. The neuroprotective effect of AMN082 was blocked by the mGlu7R NAM MMPIP, indicating that the protection conferred by AMN082 was receptor-specific.

### Traumatic brain injury (TBI)

For the first time, the neuroprotective potential of mGlu7R allosteric agonist AMN082 against TBI injury was found in the in vivo model in rats [187]. These findings demonstrated that (*ip)* administered AMN082 three times, immediately-, 24- and 48 h after TBI diminished TBI-induced cerebral cortex damage and improved posttraumatic motor impairment. However, as the authors rightly pointed out, this report has several drawbacks. One crucial drawback is the failure to verify the receptor specificity of AMN082. Since the effects of AMN082 could result from its off-target activity and its degradation into active compounds [188, 189], it is essential to conduct appropriate controls in the in vivo studies. Among others, this includes using mGlu7R-deficient (mGlu7R^−/−^) animals to ensure accurate conclusions about the involvement of mGlu7 receptors in the biological response [189].

### Neurodegenerative disorders

The neuroprotective potential of mGlu7R allosteric agonist AMN082 has been observed in experimental chronic neurodegenerative disorders, specifically in the cellular model of PD using the neurotoxin MPP(+) [175]. This study demonstrated that AMN082 provided neuroprotection against MPP(+)-induced neurotoxicity in UN-SH-SY5Y human neuroblastoma cells [175]. However, in previous studies using animal models of PD, AMN082 showed limited potential in alleviating Parkinsonian symptoms [190–192].

### mGlu8 receptor

#### Neurodegenerative disorders

The neuroprotective potential of ligands activating mGlu8R has been reported in both in vitro and in vivo studies. Jantas and colleagues demonstrated neuroprotective effects of both the orthosteric mGlu8R agonist, (*S*)-3,4-DCPG, and mGlu8R PAM, AZ12216052 against parkinsonian toxin MPP(+)-induced neurodegeneration in human neuroblastoma SH-SY5Y cells [175]. Previous research involving (*S*)-3,4-DCPG yielded mixed results, with one study demonstrating its partially positive effects on Parkinsonian-like symptoms [193], while others failed to show benefits in rat PD models [191]. Recently, Woo and colleagues reported that mGlu8R is implicated in the animal model of MS, a chronic neurodegenerative, inflammatory, and demyelinating disease of the CNS [194]. They used the model of experimental autoimmune encephalomyelitis (EAE) using wild-type mice (mGlu8R^+/+^) and mGlu8R-deficient (mGlu8R^−/−^) mice. Interestingly, the disease course of EAE was more severe in mice lacking mGlu8R than in wild-type mice. Neuroprotective effects of mGlu8R PAM AZ12216052 were observed in wild-type mice but not in mGlu8R-deficient mice. Additionally, their in vitro experiments demonstrated that AZ12216052 provided neuroprotection against glutamate-induced excitotoxicity in cortical cultures from wild-type mice but was ineffective in mGlu8R-deficient (mGlu8R^−/−^) cultures [194].

## Conclusions and future perspectives

This review summarizes the current knowledge of the neuroprotective potential of ligands targeting group III mGlu receptors, utilizing cellular and animal models of various CNS disorders, including acute conditions (ischemic stroke and TBI) and chronic neurodegenerative diseases (PD, AD, and MS). Group III mGlu ligands, especially those activating mGlu4 or mGlu7 receptors (ACPT-I and AMN082, respectively), exhibit neuroprotective effects when administered a delay following the onset of ischemic injury. These findings may be clinically significant because the therapeutic time window is crucial in establishing any neuroprotective compound’s potential clinical usefulness.

From a clinical standpoint, it’s important to highlight that in the in vivo ischemia model, histological and functional neuroprotection evaluations were conducted in healthy and hypertensive rats, aiming to replicate conditions seen in stroke patients with hypertension. In both normotensive rats and rats with essential hypertension, ACPT-I reduced the MCAO-induced cortico-striatal damage also after delayed treatment. Additionally, it is clinically significant that ACPT-I improved postischemic gait disturbances in both normotensive- and hypertensive rats because restoring and enhancing gait function is a primary objective of post-stroke rehabilitation.

Group III mGluR allosteric ligand, AMN082, provided neuroprotection in the in vitro ischemic neurodegeneration and in vivo in the TBI in rats. From a mechanistic point of view, these are interesting results because the pathological processes underlying ischemic stroke are similar to those of TBI [21]. Nevertheless, to fully assess the role of mGlu7R in neuroprotection in these acute pathological conditions, there is a need to perform experiments using more selective ligands acting at mGlu7R because the off-target activity of AMN082 and its degradation into active compounds limits its usefulness as a pharmacological tool.

mGlu4R, for its anatomical distribution and function, seems to be an attractive pharmacological target for treating PD. Despite the beneficial effects of mGlu4R PAMs in preclinical studies of PD, not only in rodents but also in non-human primates, recent clinical trials of a randomized, double-blind, controlled phase II study using foliglurax were ineffective in reducing L-DOPA-induced dyskinesia in PD patients. Nevertheless, mGlu4R PAMs demonstrate neuroprotective effects in preclinical models of PD pathology, suggesting their potential use in early-stage PD to promote neuronal repair and potentially delay the loss of DA neurons.

Due to its anatomical distribution and function, mGlu8R appears to be a promising pharmacological target for treating neuroinflammatory diseases like MS. However, to fully assess the neuroprotective potential of mGlu8R PAM, there is a need to discover more selective ligands acting at mGlu8R because the functional cross-reactivity of AZ12216052 with mGlu4R and mGlu5R limits its usefulness as a mGlu8R PAM.

Despite extensive research on neuroprotection, bridging the gap from preclinical studies to successful clinical trials remains challenging. In the context of mGlu ligands studies, it is essential to consider the complex interactions of mGlu receptors with their subtypes and other GPCR family proteins, which have been described in recent years. Notably, the possibility of forming homo- and heterodimers, which may differ in function and pharmacology depending on their protomer composition, should be emphasized. The diversity of such systems and their functionality are not considered in most current studies. To a large extent, the prevailing viewpoint still considers these receptors as simple, single sensors activated by glutamate.

In further research exploring the neuroprotective properties of mGluR ligands, it is crucial to consider not only their selectivity for specific receptor subtypes but also the protection of neurons, astrocytes, and blood vessels. Lyden and colleagues [195] suggest replacing the term “neuroprotection” with “cerebroprotection” in preclinical studies to emphasize safeguarding the entire neurovascular unit, including astrocytes (glioprotection) and blood vessels (vasculoprotection). Before clinical trials, preclinical evaluation should involve subjects of varying ages, including older animals of both sexes, and with concurrent comorbidities like hypertension, diabetes, and various metabolic disorders. Adopting these updated definitions, innovative methodologies, and a heightened focus on rigor could enhance the potential for effective cerebroprotective therapy.

## Data Availability

No datasets were generated or analysed during the current study.

## Abbreviations

(1 S,3R)-ACPD: (1 S,3R)-1-Aminocyclopentane-1,3-dicarboxylic acid.
ACPT-I: (1 S,3R,4 S)-1-aminocyclopentane-1,2,4-tricarboxylic acid.
AD: Alzheimer’s disease.
Alzheimer’s disease: (6-(2,4-dimethylphenyl)-2-ethyl-6,7-dihydrobenzo[d]oxazol-4(5 H)-one).
ago-PAMs: ago-PAMs: ago-allosteric modulators;
ALX-171: (2-(2-Chlorophenyl)-6-(2,3-dimethoxyphenyl)-3-methylquinazolin-4(3 H)-one).
AMN082: N, N-dibenzhydrylethane-1,2-diamine dihydrochloride.
AMPA: 2-amino-3-(3-hydroxy-5-methylisoxazol-4-yl) propionate.
AZ012216052: (2-[[(4-Bromophenyl)methyl]thio]-N-[4-(1-methylpropyl)phenyl]acetamide).
BBB: Blood-brain barrier.
β-arrs: β-arrestins.
Ca^2+^: Calcium ions.
cAMP: Cyclic adenosine monophosphate.
Cl⁻: Chloride ions.
CNS: Central nervous system.
CPCCCEt: 7-hydroxyiminocyclopropan[b]chromen-1a-carboxylic acid ethyl ester.
CPPG: (RS)-alpha-cyclopropyl-4-phosphonophenylglycine.
CRD: Cysteine-rich domain.
CVN636: (S)-2-(4-fluorophenyl)-N-((3 S,4 S)-4-(methylsulfonyl)chroman-3-yl)propenamide.
DAG: Diacylglycerol.
EAE: Experimental autoimmune encephalomyelitis.
GABA: Gamma-aminobutyric acid.
Glu: Glutamate.
GP: Globus pallidus.
GPCRs: G protein-coupled receptors.
GRKs: GPCR kinases; HTS, high-throughput screening.
iGluRs: Ionotropic glutamate receptors.
IP_3_: Inositol-1,4,5-triphosphate.
Ip: Intraperitoneal.
JNK3: c-Jun N-terminal kinase 3.
KA: Kainate.
L-AP4: L-2-amino-4-phosphonobutyric acid.
L-CCG: ((1 S,1′S,2′S)-carboxycyclopropylglycine).
L-SOP: L-serine-O-phosphate.
LSP1-2111: [((3 S) − 3-Amino-3-carboxy)propyl][(4-hydroxy-5-methoxy-3-nitrophenyl)hydroxymethyl]phosphinic acid.
LSP4-2022: (2 S) − 2-amino-4-({[4-(carboxymethoxy)phenyl](hydroxy)methyl} (hydroxy)phosphoryl)butanoic acid.
LU AF21934: ((1 S, 2R)-N -(3,4-dichlorophenyl)-cyclohexane-1,2-dicarboxamide).
LY341495: ((2 S)-2-Amino-2-[(1 S,2 S)-2-carboxycycloprop-1-yl]-3-(xanth-9-yl) propanoic acid).
MAP4: 2-amino-2-methyl-4-phosphonobutyrate.
MAPK/ERK: Mitogen-activated protein kinase/extracellular signal-regulated kinase.
MCPG: α-methyl-4-carboxyphenylglycine.
mGluRs: Metabotropic glutamatergic receptors.
MMPIP: (6-(4-Methoxyphenyl)-5-methyl-3-(4-pyridinyl)-isoxazolo[4,5-c]pyridin-4(5 H)-one).
MPEP: N-Phenyl-7-(hydroxyimino)cyclopropa[b]chromen-1a-carboxamide.
MPP(+): 1-methyl-4-Phenylpyridinium ion.
MPPG: (RS)-α-methyl-4-phosphonophenylglycine glycine.
MPTP: 1-methyl-4-phenyl-1,2,3,6-tetrahydropyridine.
MS: Multiple sclerosis.
MSOP: a-methyl-serine-O-phosphate.
NAMs: Negative allosteric modulators.
NMDA: N-methyl-D-aspartate.
6-OHDA: 6-hydroxydopamine.
PAMs: Positive allosteric modulators.
PI3-K/Akt: Phosphatidylinositol-3-kinase/Akt.
PD: Parkinson’s disease.
PCEP: 3-amino-3-carboxypropyl-20-carboxyethylphosphinic acid.
PHCCC: N-Phenyl-7-(hydroxyimino)cyclopropa[b]chromen-1a-carboxamide.
PSAT: Phosphoserine aminotransferase.
PSP: phosphoserine phosphatase.
PXT002331: N-{6-[3-(morpholin-4-yl)propyl]-2-(thieno[3,2-c]pyridin-6-yl)-4 H-1-benzopyran-4-ylidene}hydroxylamine.
ROS: Reactive oxygen species.
(*R*,* S*)-PPG: 4-phosphonophenylglycine.
(*S*)-3,4-DCPG): (S)-3,4-Dicarboxyphenylglycine.
SH-SY5Y: Human neuroblastoma.
SNpc: Substantia nigra pars compacta.
SNpr: Substantia nigra pars reticulata.
TBI: Traumatic brain injury.
TH-ir: Tyrosine hydroxylase immunoreactive.
TMD: Transmembrane helical domain.
VFD: Venus Flytrap Domain.
VU0155041: cis-2-[[(3,5Dichlorophenyl)amino]carbonyl]cyclohexanecarboxylic acid.
VU0361737: N-(Chloro-3-methoxyphenyl)-2-picolinamide.
VU6005649: 3-(2,3-Difluoro-4-methoxyphenyl)-2,5-dimethyl-7-(trifluoromethyl)pyrazolo[1,5-a]pyrimidine.
XAP044: 7-hydroxy-3-(4-iodophenoxy)-4 H-chromen-4-one.
